# Engineering Cell-Derived Nanovesicles for Targeted Immunomodulation

**DOI:** 10.3390/nano13202751

**Published:** 2023-10-12

**Authors:** Adil Ali Sayyed, Piyush Gondaliya, Irene K. Yan, James Carrington, Julia Driscoll, Anuradha Moirangthem, Tushar Patel

**Affiliations:** Departments of Transplantation and Cancer Biology, Mayo Clinic, Jacksonville, FL 32224, USA

**Keywords:** cell-derived nanovesicles, RNA therapeutics, immunotherapy, biological nanoparticles, targeted delivery

## Abstract

Extracellular vesicles (EVs) show promise for targeted drug delivery but face production challenges with low yields. Cell-derived nanovesicles (CDNVs) made by reconstituting cell membranes could serve as EV substitutes. In this study, CDNVs were generated from mesenchymal stem cells by extrusion. Their proteomic composition, in vitro and in vivo toxicity, and capacity for loading RNA or proteins were assessed. Compared with EVs, CDNVs were produced at higher yields, were comprised of a broader range of proteins, and showed no detrimental effects on cell proliferation, DNA damage, or nitric oxide production in vitro or on developmental toxicity in vivo. CDNVs could be efficiently loaded with RNA and engineered to modify surface proteins. The feasibility of generating immunomodulatory CDNVs was demonstrated by preparing CDNVs with enhanced surface expression of PD1, which could bind to PD-L1 expressing tumor cells, enhance NK and T cell degranulation, and increase immune-mediated tumor cell death. These findings demonstrate the adaptability and therapeutic promise of CDNVs as promising substitutes for natural EVs that can be engineered to enhance immunomodulation.

## 1. Introduction

Extracellular vesicles (EVs) have shown tremendous promise as targeted therapeutic delivery vehicles in diverse diseases, including cancer [1]. Indeed, large-scale biomanufacturing and clinical trials of cell-secreted EV-based therapies have been initiated [2]. However, the clinical translation of EVs faces substantial obstacles, such as low and variable yields, difficulties in surface engineering, and batch-to-batch inconsistencies [3]. These limitations have motivated the development of alternative approaches to generating effective EV-based therapeutics.

Cell-derived nanovesicles (CDNVs) are artificially generated vesicles that are generated from cells and retain membrane proteins and biological activity [4,5]. These EV-mimetics can be produced in higher yields by methods like mechanical extrusion, microfluidics, or sonication [6,7,8,9]. As CDNVs have similarities in size distribution, shape, and membrane proteins with endogenously produced EVs, they are attractive alternatives to EVs as therapeutic delivery vehicles. Like EVs, CDNVs can be loaded with therapeutic proteins, small molecules, or nucleic acids. CDNVs may retain the beneficial properties of natural EVs while overcoming challenges faced by EVs, such as manufacturing scalability and ease of modification of their surface or content [10,11,12]. As they are derived from cell membranes, CDNVs retain membrane proteins present in their cells of origin. This provides the opportunity for the production of vesicles that express specific surface molecules for targeting or biological activity through cellular engineering of their parental cells [13,14]. CDNVs can also be directly engineered, for example, by hybridization with liposomes for drug delivery [10]. Despite these potential advantages, comprehensive analyses of CDNV toxicity, cargo loading capacity, and the ability of surface engineering to confer targeting specificity remain lacking. Elucidating these parameters is critical to evaluating and optimizing the potential of CDNVs as adaptable and scalable platforms for targeted nanotherapies. 

Therefore, this study sought to conduct rigorous in vitro and in vivo safety assessments of CDNVs, determine their loading efficacy for therapeutic RNA/proteins, and demonstrate surface engineering with enhanced expression of immunomodulatory proteins. The findings provide foundational support for the development of CDNVs as next-generation biological nanotherapies tailored for clinical applications.

## 2. Materials and Methods

### 2.1. Cell Isolation and Culture

Human mesenchymal stem cells (hMSCs) were purchased from Lonza (Basel, Switzerland). Human adipose-derived MSCs (A-MSCs), HepG2, HL-60, HEK293T, and RAW264.7 cells were purchased from ATCC (Manassas, VA, USA), and human hepatocytes (HH) were purchased from ScienCell (Carlsbad, CA, USA). HuCCT1 human cholangiocarcinoma (CCA) cells were provided by Dr. Copland (Mayo Clinic). MSCs and HH were cultured in the manufacturer’s suggested media. HepG2, HuCCT1, and HEK-293T cells were cultured in Dulbecco’s Modified Eagle Medium-High Glucose (DMEM-HG, Cytivia, Marlborough, MA, USA) supplemented with 10% fetal bovine serum (FBS) and 1% penicillin–streptomycin (Gemini Bio, Sacramento, CA, USA). HL-60 cells and RAW264.7 cells were cultured in RPMI 1640 media (Gibco, Billings, MT, USA) supplemented with 10% FBS and 1% penicillin–streptomycin. For immune cell isolations, human whole blood was obtained from the Department of Transfusion Medicine at Mayo Clinic, and primary peripheral blood mononuclear cells (PBMC) were extracted using Lympholyte-H (Cedarlane Labs, Burlington, ON, Canada) [4]. Natural killer (NK) cells were isolated from PBMC using a human NK cell isolation kit (Miltenyi Biotec, Bergisch Gladbach, Germany) and propagated in RPMI 1640 medium (Corning Inc., Corning, NY, USA) supplemented with 10% FBS, 500 IU/mL IL-2 (Roche Diagnostics, Indianapolis, IN, USA), and 140 IU/mL IL-15 (R&D Systems, Minneapolis, MN, USA) for 14 days. T-cells were isolated from PBMC using a human CD8+ T cell isolation kit (Miltenyi Biotec, Bergisch Gladbach, Germany), activated with human anti-CD3 and human anti-CD8 (Thermo Fisher Scientific, Waltham, MA, USA) monoclonal antibodies, and grown in RPMI 1640 medium supplemented with 10% FBS and 500 IU/mL IL-2.

### 2.2. Generation and Characterization of EVs and CDNVs

EVs were isolated by differential ultracentrifugation as previously described [15]. CDNVs were isolated from A-MSCs or hMSCs by cell extrusion. Cells were washed with phosphate-buffered saline (PBS) and then incubated with PBS containing protease inhibitor (PBS-PI, 1 mg/mL) for 10 min. Cells were then scrapped off gently using PBS-PI. The cell suspension was sequentially passed through decreasing pore-sized membranes with 10, 5, 1, 0.4, and 0.1 µm sized pores (T&T Scientific Corp., Knoxville, TN, USA), followed by centrifugation at 2000× *g* for 10 min at 4 °C. The supernatant was collected, and ultracentrifugation was performed at 100,000× *g* for 70 min at 4 °C. The pellet containing CDNVs was resuspended in PBS. Nanoparticle tracking analysis (NTA) was used to characterize the CDNVs (NanoSight NS300, Malvern Panalytical, Malvern, UK).

### 2.3. Proteomic Analysis of EVs and CDNVs

Proteomic analysis was performed at the Mayo Clinic Proteomics Core (Rochester, MN, USA). In brief, protein concentration was measured using a bicinchoninic acid assay (BCA) kit (Thermo Fisher Scientific, Waltham, MA, USA), and 20 µg of sample was subjected to electrophoresis on a 10.5–14% criterion gel. Subsequently, the gel was divided into eight sections based on molecular weight, LC–MS/MS analysis was performed, and peptides were identified.

### 2.4. Cytotoxicity Assay

Cells were seeded in a 96-well plate at a density of 10,000/well and allowed to attach overnight prior to incubation with PBS or CDNVs. After 72 h, cell viability was assessed using an MTS assay (Promega, Madison, WI, USA). For each experimental condition, viability was expressed as a percent of the viable cell count in the untreated control group.

### 2.5. Nitric Oxide (NO) Assay

RAW264.7 cells were seeded in a 96-well plate at a density of 20,000/well and allowed to attach overnight. They were incubated with CDNVs, 10 ng/mL lipopolysaccharide (positive control), or PBS (negative control) for 24 h. NO production by macrophages in the presence of CDNVs was quantitated using the Total Nitric Oxide and Nitrate Parameter Assay Kit (R&D Systems).

### 2.6. DNA Damage Assay

HL-60 cells were treated with CDNVs, 20 µM Etoposide (positive control), or PBS (negative control) for 24 h at 37 °C. The cells were subsequently harvested, and DNA damage was assessed using the OxiSelect comet assay (Cell Biolabs Inc., San Diego, CA, USA). Images were captured using an EVOS M5000 microscope (Thermo Fisher Scientific, Waltham, MA, USA), and tail length was calculated using the OpenComet software v1.3.1 (CometBio, Chicago, IL, USA) in ImageJ software v1.53t (NIH, Bethesda, MD, USA).

### 2.7. Assessment of Developmental Toxicity

Zebrafish (Danio rerio) were obtained from Dr. Leonard Zon and housed in the Mayo Clinic Jacksonville zebrafish facility under an Institutional Animal Care and Use Committee (IACUC)-approved protocol (A00003654-18). The zebrafish were fed live brine shrimp (Artemia nauplii) twice a day and dry flakes (pellets) (Zeigler Bros. Inc., Gardners, PA, USA) once a day. Fertilized embryos were collected, rinsed with embryo water (EW, 5 nM NaCl, 0.17 nM KCl, 0.33 nM CaCl_2_, 0.33 MgSO_4_, 0.00001% methylene blue), transferred to single wells of a 96-well plate, maintained in 100 µL EW for 24 h, and subsequently treated with EW, vehicle (PBS), or CDNVs every day for up to 7 days post fertilization (dpf) as previously described [16]. Dechorionation was measured between 45 and 75 h post-fertilization (hpf). The heart rates of zebrafish were recorded at 2, 3, 6, and 7 dpf. Developmental toxicity was assessed by examining for malformations, developmental delay by assessing time to hatching, and cardiac effects by assessing heart rate throughout the study. 

### 2.8. RNA and Protein Loading

The RNA loading efficiency of CDNVs was determined using a ribogreen assay. A total of 250 pmol of ribogreen dye-labeled oligonucleotide in 25 µL of OptiMEM media (Gibco) was mixed with 4 µL of Lipofectamine 2000 (Thermo Fisher Scientific, Waltham, MA, USA) in 25 µL of OptiMEM media and incubated for 10 min at room temperature (RT). The mixture was then added to an equal volume of CDNVs and incubated for 30 min at RT, followed by filtration using an Amicon Ultra 10 kDa centrifugal filter unit (MilliporeSigma, Burlington, MA, USA). The pellets containing CDNVs were resuspended in PBS, and fluorescence was measured at excitation 480 nm/emission 520 nm using a FLUOstar Omega microplate reader (BMG Labtech, Cary, NC, USA). Oligonucleotide loading was calculated using a standard curve generated from known concentrations of labeled oligonucleotides. To assess ribonucleoprotein (RNP) loading efficiency, Cas9 mRNA (TriLink BioTechnologies, San Diego, CA, USA) was mixed with guide RNA (Synthego Corporation, Redwood City, CA, USA) in a 5:1 ratio in NEB buffer and incubated at 25 °C for 10 min to form RNP complexes. RNP (2.5 µg) was then mixed with 10 µL of Cas9 plus reagent (Thermo Fisher Scientific, Waltham, MA, USA) in 50 µL of optiMEM in one tube, while 6 µL of Lipofectamine CRISPRMAX Cas9 reagent (Thermo Fisher Scientific, Waltham, MA, USA) was mixed in 50 µL of optiMEM in another tube. The contents of both tubes were then mixed at RT for 10 min, added to 1 × 10^11^ CDNVs, and incubated at 4 °C for 10 min, followed by ultracentrifugation at 100,000× *g* for 70 min at 4 °C. The pellet was resuspended in RIPA Lysis and Extraction Buffer (Thermo Fisher Scientific, Waltham, MA, USA), and the loading efficiency was determined by quantitative immunoblot analysis [17]. 

### 2.9. Protein Extraction and Western Blot

Total proteins were extracted using RIPA buffer and quantified by the Pierce BCA Protein Assay Kit. The protein was separated on 4–12% NuPAGE Bis-Tris gels (Thermo Fisher Scientific, Waltham, MA, USA) and transferred to a nitrocellulose membrane using an iBlot-2 system (Thermo Fisher Scientific, Waltham, MA, USA). The membrane was blocked for 1 h at RT using Odyssey blocking buffer (LI-COR Biosciences, Lincoln, NE, USA) and incubated with primary antibody overnight at 4 °C, followed by incubation with secondary antibody for 1 h at RT. The membrane was washed with Tween containing PBS (PBST) before imaging under the Odyssey Imaging System (LI-COR Biosciences, Lincoln, NE, USA). The expression of proteins was normalized with the endogenous control GAPDH, and the densitometric analysis was conducted using ImageJ software v1.53t. 

### 2.10. Virus Production and Transduction

Lentiviral particles were generated by transfecting target plasmids with envelope (pMD2.G) and packaging (psPAX2) plasmids into HEK293T cells using Lipofectamine 2000 in an antibiotic-free DMEM-HG medium. After transduction, the medium was replaced with a fresh DMEM-HG medium, and viral particles were collected after 48 h. To transduce A-MSCs, 1 million cells were incubated overnight with 5 mL of pHR-PD1-mGFP or pHR-mGFP virus supernatant mixed with 5 mL of MSC-specific medium with 10 µg/mL polybrene (MilliporeSigma, Burlington, MA, USA). The expression of PD1 and mGFP on MSCs was confirmed by Western blot and fluorescent microscopy, respectively. Similarly, HuCCT1 cells were transduced with GFP-Luciferase virus particles in the DMEM-HG medium. GFP-Luciferase-positive HuCCT1 cells were sorted by the Mayo Clinic Jacksonville Research core facility of Imaging and Flow Cytometry using a cell sorter (BD Biosciences, Franklin Lakes, NJ, USA), and luciferase expression was confirmed by measuring the luminescence intensity using an IVIS spectrum imaging system (PerkinElmer, Inc., Waltham, MA, USA).

### 2.11. Generation of PD1+ CDNVs

To generate PD1+ CDNVs, PD1-expressing A-MSCs were used, and the CDNVs were extracted using the same protocol described above. PD1 expression on CDNVs was confirmed by the ImageStreamX MKII imaging flow cytometer (Millipore Sigma, Burlington, MA, USA) [18]. CDNVs were first stained with lipophilic dye DiO (Thermo Fisher Scientific, Waltham, MA, USA) by incubating 1 µM of dye with CDNVs at 37 °C for 20 min and labeled CDNVs were collected by ultracentrifugation as mentioned above. DiO labeled CDNVs were then incubated with rabbit anti-human PD1 antibody (Cell Signaling Technology, Danvers, MA, USA) for 1 h at RT, followed by incubation with anti-rabbit AlexaFlour647 secondary antibody (Thermo Fisher Scientific, Waltham, MA, USA) in PBS for 1 h at RT. The labeled CDNVs were washed and collected using ultracentrifugation. The stained CDNV samples were imaged at 60× magnification with slow speed and high sensitivity while acquiring data on Ch01 for Brightfield, Ch02 for DiO, and Ch05 for AlexaFlour647. Appropriate controls, single-color stains, and calibration beads were used to adjust spectral compensation. A total of 5000 events were acquired for each sample using INSPIRE software v99.4.437.0 (MilliporeSigma, Burlington, MA, USA). Data were analyzed using IDEAS software v6.2 (MilliporeSigma, Burlington, MA, USA) and FlowJo v10.8.1 (BD Biosciences, Franklin Lakes, NJ, USA).

### 2.12. PD1+ CDNV Binding to Tumor Cells

HuCCT1 cells (2000 cells/well) were seeded in a Nunc Lab-Tek II chamber slide (Thermo Fisher Scientific, Waltham, MA, USA) and allowed to attach overnight. The following day, the cells were treated with 1 × 10^9^ control or PD1+ CDNVs for 3 h on ice, and the cells were gently washed with PBS and blocked with Odyssey blocking buffer for 1 h at RT. Cells were then incubated with an anti-PD-L1-APC antibody for 1 h at RT and then stained with 1 µg/mL DAPI (Thermo Fisher Scientific, Waltham, MA, USA) for 10 min at RT and again washed with PBST. The samples were fixed with 2% paraformaldehyde for 10 min at RT and mounted using AquaPoly Mount (Polysciences, Warrington, PA, USA). Images were captured using a confocal microscope (LSM880, Zeiss, Oberkochen, Germany). 

### 2.13. PD1+ CDNV Uptake in Tumor Cells

HuCCT1 cells were seeded in a 6-well plate at a density of 250,000/well and allowed to attach overnight. Cells were then treated with DiO-labeled 1 × 10^10^ control or PD1+ CDNVs for 6 h, washed with PBS, and then harvested and stained with Live-or-Dye 568/583 (Biotium, Fremont, CA, USA). Uptake was assessed using an ImageStream X MKII imaging flow cytometer with the following settings: 60× magnification, slow flow speed, high sensitivity, and the “remove beads” option unselected. A total of 10,000 events were acquired for each sample. The raw data were compensated using the IDEAS software v6.2, and the data were exported as .fcs files and further analyzed in FlowJo v10.8.1. 

### 2.14. Biodistribution of PD1+ CDNVs

Biodistribution was assessed in 6-week-old male NSG mice provided by Dr. Qin Hong (Mayo Clinic) and housed in individually vented cages (Allentown Inc., Allentown, NJ, USA) with bed-o’cobbs bedding (The Andersons, Maumee, OH, USA). Animal studies were conducted under a Mayo Clinic IACUC protocol (A00002125-16-R22). The mice were fed food (Pico Diet 5053, LabDiet, St. Louis, MO, USA) and water ad libitum and maintained in a 12 h/12 h light/dark cycle. To generate an orthotopic intrahepatic tumor model, 2 × 10^6^ HuCCT1-GFP-Luc cells were injected into the left lobe of the mice’s liver. IVIS imaging was performed weekly to monitor tumor growth. Mice were intravenously injected with DiR (Thermo Fisher Scientific, Waltham, MA, USA) labeled 1 × 10^10^ control or PD1+ CDNVs. After 6 h, organs were collected, and bioluminescence was quantitated using an IVIS imager. 

### 2.15. Flow Cytometry Assay

Flow cytometry was performed using a NovoCyte flow cytometer (ACEA Biosciences, Inc., San Diego, CA, USA). For PD1 expression analyses, mGFP or PD1-mGFP transduced MSCs were labeled with anti-PD1-APC antibodies (Miltenyi Biotec, Bergisch Gladbach, Germany). For CD107a expression analysis, HuCCT1 cells were pretreated with 1 × 10^10^ control CDNVs and PD1+ CDNVs for 3 h, followed by co-culture with T cells or NK cells for 6 h at a 5:1 or 10:1 effector to target (E:T, immune cell: HuCCT1 cell) ratio in the presence of GolgiStop (BD Bioscience, Franklin Lakes, NJ, USA) and anti-CD-107a-APC antibody (BD Bioscience, Franklin Lakes, NJ, USA) in RPMI 1640 medium. The immune cells were collected and stained with anti-CD8-FITC or anti-CD56-FITC (Miltenyi Biotec, Bergisch Gladbach, Germany) for NK and T cells, respectively, prior to flow cytometry. Cytometry data were analyzed using FlowJo v10.8.1. 

### 2.16. T Cell- or NK Cell-Mediated Cytotoxicity

A total of 5000 HuCCT1-GFP-Luc cells were seeded in a 96-well plate and allowed to attach overnight. On the following day, cells were pretreated with 1 × 10^10^ control CDNVs or PD1+ CDNVs for 3 h and then co-cultured with primary human CD8+ T cells or NK cells for 6 h at a 5:1 or 10:1 E:T ratio for 24 h. Luciferase activity was measured using the FLUOstar Omega microplate reader. 

### 2.17. Statistical Analysis

All studies were performed in multiple replicates. Data are reported as the mean, standard error of the mean, or standard deviation as described. Differences between groups were compared using the Student’s *t*-test, and a one-way ANOVA was used to compare three or more groups. Toxicity was determined by survival analyses, and the data were analyzed by Kaplan–Meier analysis and compared between groups using a log-rank test with Bonferroni correlation. Analyses were performed using GraphPad Prism v9.5.0 (GraphPad Inc., San Diego, CA, USA). *p* < 0.05 was considered significant.

## 3. Results

### 3.1. Generation of CDNVs

**Figure 1:**
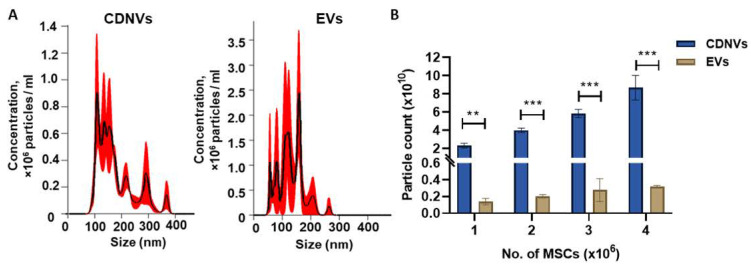
Characterization of cell-derived nanovesicles (CDNVs). CDNVs were generated using mechanical extrusion, while extracellular vesicles (EVs) were isolated using differential ultracentrifugation from adipose-derived mesenchymal stem cells (A-MSCs). (**A**) The concentration and size were assessed using nanoparticle tracking analysis. (**B**) The yield of CDNVs and EVs was assessed at different starting concentrations of cells. The values are expressed as the mean ± standard deviation (n = 3 replicates). Statistically significant data were represented as follows: (**) *p* < 0.01, (***) *p* < 0.001.

Nanovesicles were obtained from low passage MSCs by serial extrusion. This method resulted in the rapid production of a large number of CDNVs with an average size of 177.3 ± 2 nm, consistent with that of cell-derived EVs (Figure 1A). However, the yield of CDNVs was higher, with 1 × 10^6^ cells/mL yielding 1 × 10^10^ CDNVs, and considerably higher than that of EVs isolated from identical numbers of cells by sequential ultracentrifugation [19]. The yield of CDNVs generated increased proportionately with the starting concentration of cells but was limited beyond 4 × 10^6^ cells due to membrane pore clogging (Figure 1B). Scale-up beyond analytical laboratory scale generation may be feasible with starting volume or technical modifications, but they were not systematically examined.

### 3.2. Proteomic Characterization of CDNVs

**Figure 2:**
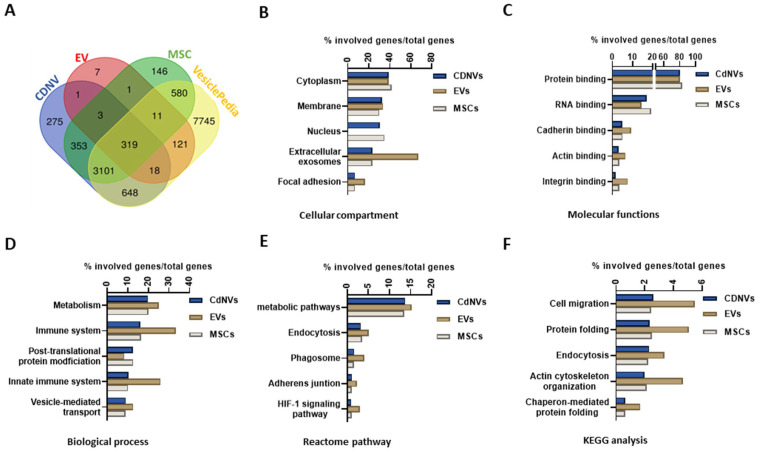
Proteomic characterization of CDNVs. (**A**) Venn diagram represents the number of distinct proteins identified in CDNVs, EVs, and MSCs that are reported in the vesiclepedia database. Gene ontology (GO) analysis of identified proteins from CDNVs, EVs, and MSCs samples with gene enrichments by (**B**) cellular compartment, (**C**) molecular functions, (**D**) biological process, (**E**) Reactome pathway, and (**F**) KEGG analysis. Protein enrichment is represented as the percent of hits on total genes identified.

The protein composition of CDNVs was examined. Mass spectrometry analysis revealed 4813 proteins in CDNVs, 508 in EVs, and 4605 in parental MSCs. Amongst these, 341 proteins were shared between CDNVs and EVs, while 4377 proteins were specific to CDNVs, and 140 were unique to EVs. A total of 3454 proteins were present in CDNVs and MSCs but not in EVs, emphasizing a distinctive protein composition of CDNVs similar to that of the parental cells (Figure 2A). The vast majority of proteins identified in CDNVs matched (4086 and 4581, respectively) with vesicle-associated proteins within the EV content database Vesiclepedia version v4.1 (http://microvesicles.org/ accessed on 31 July 2023) [20]. 

Gene ontology (GO) analysis revealed a common enrichment of proteins in the cytoplasm and membrane among CDNVs, EVs, and MSCs. However, EVs showed an absence of proteins enriched in the nucleus, unlike CDNVs and MSCs. Compared with CDNVs and MSCs, EVs had a high enrichment of proteins associated with extracellular exosomes, as well as proteins associated with focal adhesion (Figure 2B). Enrichment of proteins involved in protein binding and RNA binding, as well as enrichment of proteins involved in metabolism, post-translation protein modification, and vesicle-mediated transport, were identified in all sample types (Figure 2C,D). Proteins involved in metabolic pathways and endocytosis were similarly enriched among CDNVs, EVs, and MSCs (Figure 2E). In comparison to EVs, CDNVs and MSCs exhibited lower enrichment of proteins involved in cell migration, protein folding, endocytosis, actin cytoskeleton organization, chaperone-mediated protein folding, as well as proteins related to the immune system and innate immune system (Figure 2D–F).

### 3.3. In Vitro Safety Assessments 

**Figure 3:**
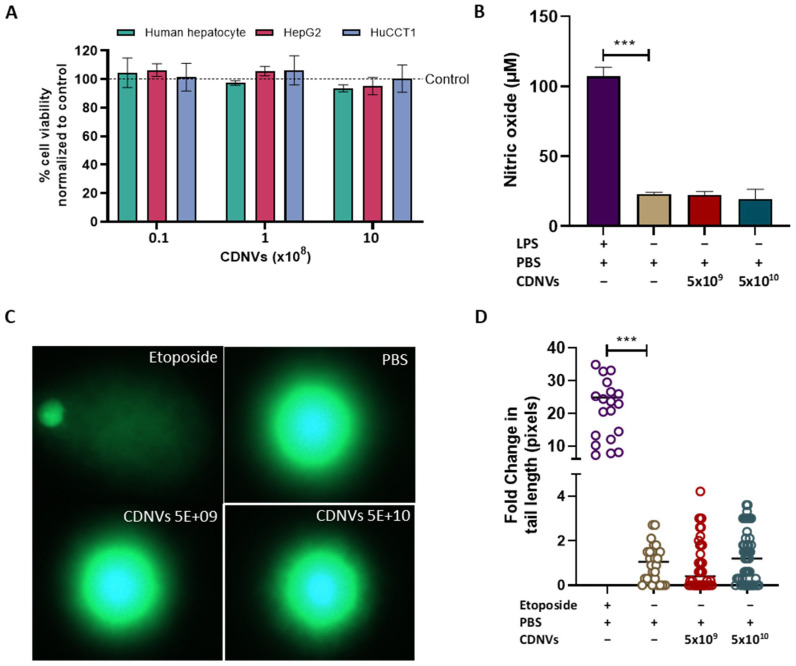
In vitro safety evaluation of CDNVs. (**A**) Human hepatocytes (HH), HepG2, and HuCCT1 cells were treated with 1 × 10^7^, 1 × 10^8,^ or 1 × 10^9^ particles of CDNVs for 24 h, and cell proliferation was assessed by MTS assay relative to the untreated control. HL-60 cells and RAW264.7 macrophages were treated with 5 × 10^9^ or 5 × 10^10^ CDNVs. At 24 h post-treatment, (**B**) nitric oxide (NO) release from macrophages was assessed by a nitrite assay, and (**C**,**D**) DNA damage in HL-60 cells was evaluated using a comet assay. The values are expressed as the mean ± standard deviation (n = 3 replicates). Statistically significant data were represented as follows: *** *p* < 0.001.

Rigorous in vitro and in vivo safety studies using relevant models can provide essential insights into the safety of CDNVs that are needed to guide appropriate dose selection and inform risk-versus-benefit analysis. As artificially generated constructs, the biocompatibility profile of CDNVs remains poorly defined compared to endogenous EVs or other nanoparticles. Although prepared from natural cell sources, their production methods could impart toxic effects. We performed assessments of the potential cytotoxicity and inflammatory effects of CDNVs on cultured cells in vitro. A range of human cell types were tested, including immune cells, hepatocytes, and cancer cells. Across cell lines, CDNV treatment did not impact viable cell numbers even at high doses, demonstrating a lack of cytotoxicity (Figure 3A). CDNVs did not stimulate or suppress NO production in macrophages, suggesting negligible induction of inflammation (Figure 3B). The results of studies on other immune cells are reported below. No DNA damage was observed in HL-60 cells following CDNV exposure, indicating the lack of genotoxic effects (Figure 3C,D). Together, these in vitro evaluations provide supporting evidence for the biocompatibility of CDNVs, with no concerning toxic mechanisms identified.

### 3.4. In Vivo Safety of CDNVs

**Figure 4:**
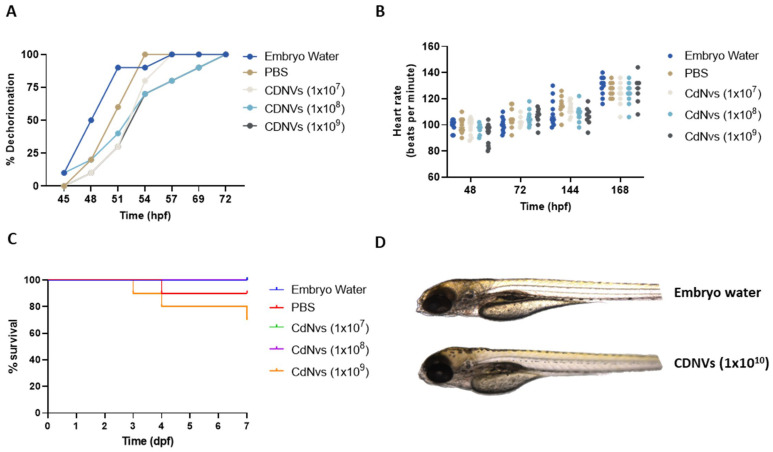
In vivo safety evaluation of CDNVs. For in vivo toxicity evaluation, fertilized zebrafish embryos were administered with 1 × 10^7^, 1 × 10^8,^ and 1 × 10^9^ PBS or embryo water (EW) every day for 7 days. (**A**) The rate of hatching was monitored starting at 48 h post-fertilization (hpf) and was recorded every 3 h until all the embryos had hatched. (**B**) The heart rate was recorded every 24 hpf until the study endpoint. (**C**) Survival was observed every day post-fertilization (dpf). (**D**) Representative images of zebrafish at 7 dpf. The values are expressed as the mean ± standard deviation (n = 10 replicates).

In addition to in vitro safety assessments, the developmental toxicity of CDNVs was assessed using zebrafish models, as these offer unique advantages that make them well-suited for this purpose. As vertebrates, they share considerable genetic homology with humans, while their rapid embryonic development and transparency allow easy monitoring for defects [21]. We have previously described protocols for efficient in vivo toxicity assessment of EV preparations using zebrafish [22]. Studies in zebrafish embryos showed only a minor delay in dechorionation with CDNV exposure, which resolved by 54 hpf (Figure 4A). Transient decreases in heart rate were noted to have self-resolved over time (Figure 4B). Importantly, CDNV treatment resulted in no developmental defects or mortality compared to control groups (Figure 4C,D). The lack of embryonic toxicity supports further investigation of CDNVs as potentially safer alternatives to natural vesicles for therapeutic applications.

### 3.5. Feasibility of Payload Incorporation into CDNVs

**Figure 5:**
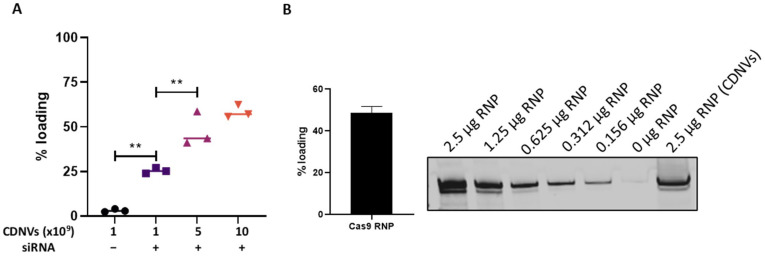
Assessment of the RNA and protein loading efficiency of CDNVs. (**A**) For RNA loading efficiency, 1 × 10^9^, 5 × 10^9^, and 1 × 10^10^ CDNVs were loaded with 250 pmol of ribogreen dye-labeled negative control sequence of siRNA oligonucleotide, and RNA loading efficiency was evaluated by measuring the fluorescence. (**B**) Ribonucleoprotein (RNP) loading efficiency was evaluated by quantifying loaded RNP using Western blot. The values are expressed as the mean ± standard deviation (n = 3 replicates). Statistically significant data were represented as follows: (**) *p* < 0.01.

Harnessing the potential of CDNVs as therapeutic delivery vehicles requires efficient loading of biologically active cargo. Thus, the ability to package RNA or proteins into CDNVs was assessed using transfection-based approaches. Incubation of CDNVs with fluorescently labeled RNA resulted in a concentration-dependent loading (Figure 5A), with encapsulation efficiency comparable to that of cell-derived EVs [23,24]. CDNVs were also able to effectively package functional Cas9 RNP complexes. Western blot quantification revealed around 50% loading efficiency of the protein cargo when 5 × 10^10^ CDNVs were loaded with 2.5 µg of RNP complex (Figure 5B). Protection of the loaded RNA and protein was confirmed by RNase degradation assays. Moreover, maintenance of RNP enzymatic activity following CDNV loading verified the retention of cargo functionality. Together, these results highlight the excellent capacity of CDNVs to incorporate diverse therapeutic cargo.

### 3.6. Generation of Engineered CDNVs

**Figure 6:**
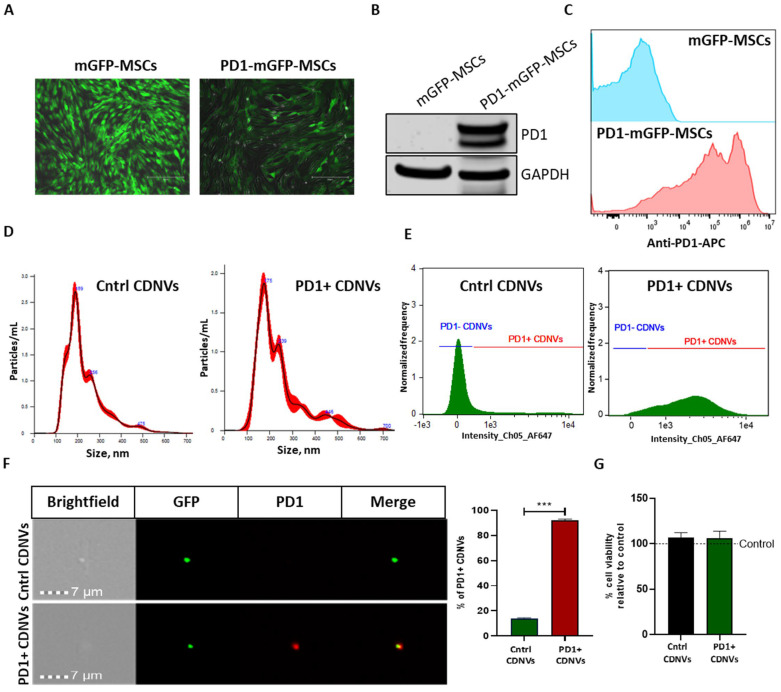
Generation and characterization of PD1-expressing CDNVs. (**A**) A-MSCs were transduced with membrane GFP (mGFP) or PD1-mGFP lentivirus. (**B**) Total PD1 expression in mGFP or PD1-mGFP transduced A-MSCs was confirmed by Western blot, and (**C**) PD1 on their surface was evaluated using flow cytometry. Control CDNVs and PD1+ CDNVs isolated from mGFP and PD1-mGFP transduced A-MSCs, respectively, were characterized for particle size by (**D**) NTA and (**E**,**F**) PD1 expression on their surface by ImageStream flow cytometry. (**G**) HuCCT1 cells were incubated with 1 × 10^9^ control of PD1+ CDNVs for 24 h, and viable cells were assessed using an MTS assay. The values are expressed as the mean ± standard deviation (n = 3 replicates). Statistically significant data were represented as follows: ***, *p* < 0.001.

Engineered CDNVs were generated from A-MSCs that were biologically modified to express PD1-mGFP (Figure 6A–C). A-MSCs were used as source cells for functional studies since these can be readily obtained in an autologous patient-specific manner from subcutaneous fat [25]. The yield and size of PD1-expressing CDNVs were similar to those of control CDNVs (Figure 6D). Surface expression of PD1 on engineered CDNVs was verified by flow cytometry data (Figure 6E,F), with ~90% of generated CDNVs expressing PD1 on their surface. The engineered CDNVs did not directly affect HuCCT1 cell viability (Figure 6G).

**Figure 7:**
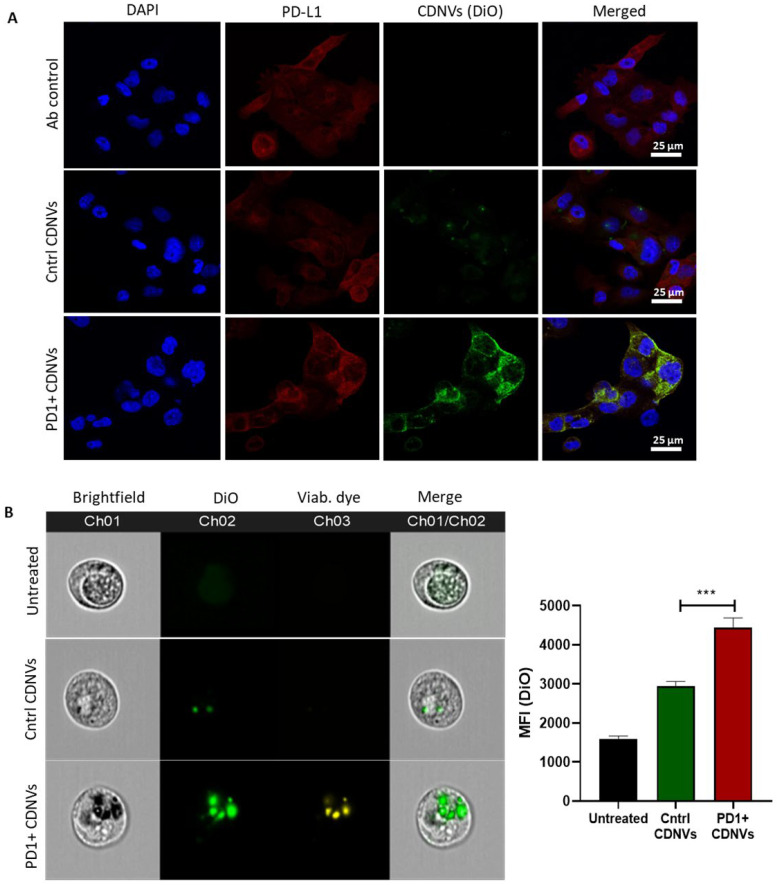
PD1-expressing CDNVs bind to PD-L1 and exhibit enhanced uptake efficiency. HuCCT1 cells were incubated with 1 × 10^9^ and 1 × 10^10^ DiO-labeled control or PD1+ CDNVs for binding and uptake studies, respectively. (**A**) The HuCCT1 cells were stained with PD-L1 antibody (AF647) and DAPI (nucleus), and images were captured using the confocal microscope; the scale bar represents 25 µm. For uptake studies, imaging-based flow cytometry was performed using ImageStream. (**B**) The DiO-stained CDNVs were detected in channel Ch02, and the viability dye was visualized in channel Ch03. IDEAS software v6.2 was used to extract .fcs files, and the mean fluorescence intensity (MFI) was calculated in FlowJo v10.8.1. The values are expressed as the mean ± standard deviation (n = 3 replicates). Statistically significant data were represented as follows: ***: *p* < 0.001.

To evaluate their binding to PD-L1 on cancer cells, HuCCT1 tumor cells were stained with a PD-L1 antibody and incubated with control or targeted CDNVs. Considerably higher affinity for binding and uptake by PD-L1 expressing cells was observed with engineered CDNVs compared with control CDNVs (Figure 7). Co-localization of labeled CDNVs with PD-L1 labeling supported the presence of interactions between PD1 and PD-L1.

### 3.7. Biodistribution of Engineered CDNVs

**Figure 8:**
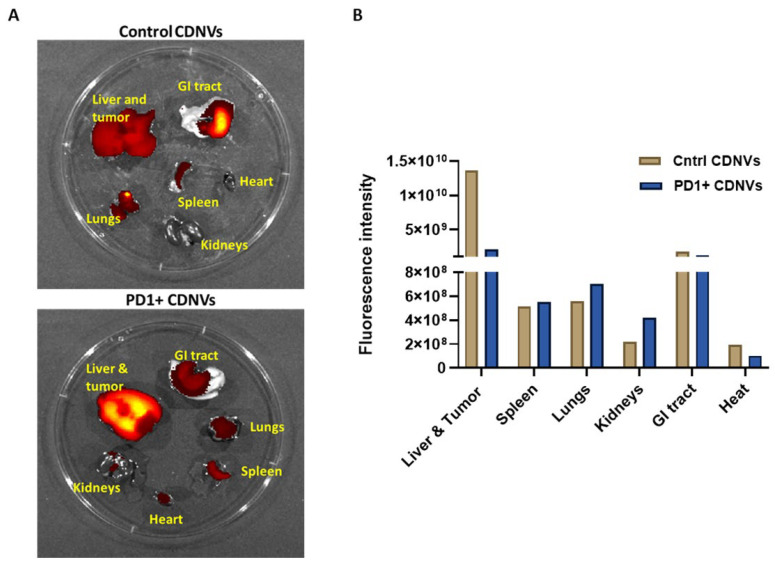
Biodistribution of PD1-expressing CDNVs. (**A**,**B**) Tumor-bearing mice were intravenously injected with DiR labeled 1 × 10^10^ control CDNVs or PD1+ CDNVs. After 6 h, organs were collected, and the fluorescence was measured ex situ using an IVIS imaging system.

We evaluated the biodistribution of CDNVs in vivo in orthotopic xenografts of HuCCT1 cells. Fluorescently labeled control or PD1+ CDNVs were intravenously administered to mice bearing orthotopically implanted tumor cell xenografts, followed by ex situ assessment of fluorescence to determine the biodistribution of administered CDNVs within isolated organs. As observed previously with EVs, there was a high biodistribution of CDNVs to the liver. In tumor-bearing livers, there was a greater uptake of PD1+ CDNVs compared with control CDNVs, consistent with enhanced uptake in the presence of tumor cells (Figure 8).

### 3.8. Engineered CDNVs-Mediated T Cell Priming

**Figure 9:**
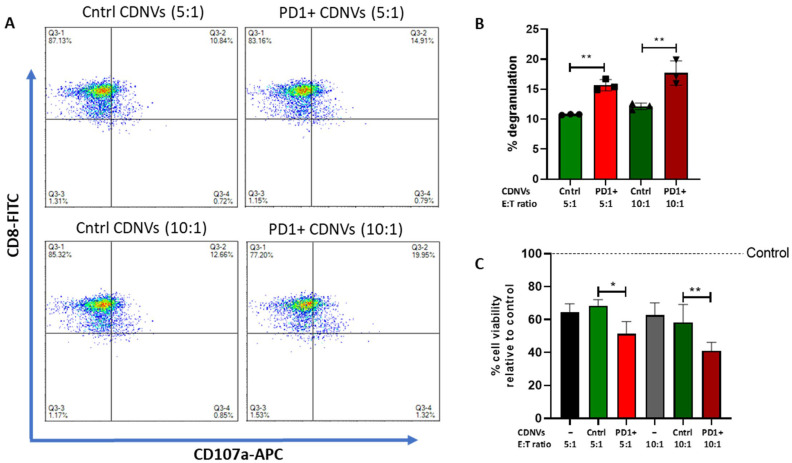
PD1-expressing CDNVs potentiate T cell-mediated cytotoxicity. HuCCT1 cells were pretreated with 1 × 10^10^ control or PD1+ CDNVs for 3 h and 6 h for degranulation and cytotoxicity experiments, respectively. CDNV pretreated HuCCT1 cells were incubated with T cells in an effector: target (E:T) ratio of 5:1 or 10:1 to assess (**A**,**B**) the degranulation activity of T cells and (**C**) their cytotoxicity on cancer cells, relative to untreated control. The values are expressed as the mean ± standard deviation (n = 3 replicates). Statistically significant data were represented as follows: *, *p* < 0.05; **, *p* < 0.01.

The PD1/PD-L1 axis has emerged as a promising target for modulation of immune responses by therapeutic EVs [26]. Thus, we explored the ability of engineered CDNVs to modulate immune-related biological effects. PD-L1 expressed in cancer cells can bind to PD1 on T cells and suppress their activation. We hypothesized that PD1-expressing CDNVs could bind to cancer cell PD-L1, blocking this immunosuppressive signaling and preventing tumor cell immune evasion. To test this, control CDNVs or PD1-expressing CDNVs were incubated with PD-L1-positive HuCCT1 cancer cells, then co-cultured with primary human CD8+ T cells at different E:T ratios. Pre-treatment with PD1-CDNVs increased degranulation of CD8+ T cells at both 5:1 and 10:1 E:T ratios, indicating enhanced activation (Figure 9). Moreover, this was associated with increased T cell-mediated tumor cell cytotoxicity at both 5:1 and 10:1 E:T ratios which was more pronounced at 10:1 ratio. Thus, PD1-engineered CDNVs can bind to tumor cell PD-L1 and block immunosuppressive signaling to T cells. This resulted in heightened CD8+ T cell activation and anti-tumor cytotoxicity and demonstrated the potential of tailored CDNVs to enhance T cell-mediated immune responses against cancer cells.

### 3.9. Effects of Engineered CDNVs on NK Cells

**Figure 10:**
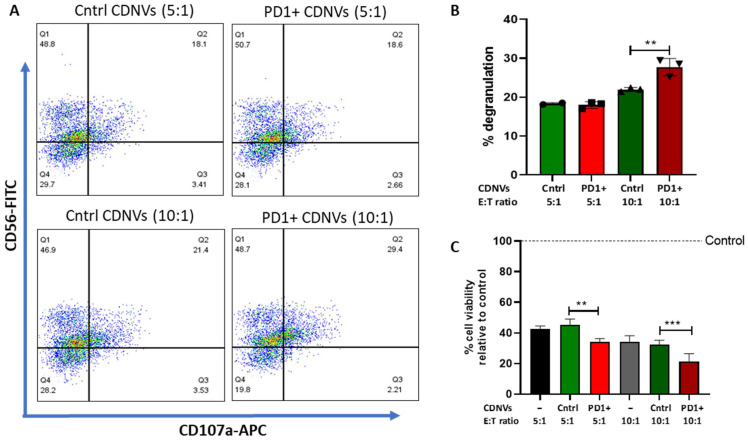
PD1-expressing CDNVs potentiate NK cell-mediated cytotoxicity. HuCCT1 cells were pretreated with 1 × 10^10^ control or PD1+ CDNVs for 3 h and 6 h for degranulation and cytotoxicity experiments, respectively. CDNV-pretreated HuCCT1 cells were incubated with NK cells in an effector to target (E:T) ratio of 5:1 or 10:1 to assess (**A**,**B**) the degranulation activity of NK cells and (**C**) their cytotoxicity on cancer cells, normalized to the untreated control. The values are expressed as the mean ± standard deviation (n = 3 replicates). Statistically significant data were represented as follows: **, *p* < 0.01; ***, *p* < 0.001.

Next, we evaluated the ability of PD1-engineered CDNVs to modulate NK cell activity by interfering with PD1/PD-L1 immune checkpoint signaling. NK cells, a component of the innate immune system, play an important role in detecting and eliminating tumor cells. However, cancer cells can evade NK cell attack by upregulating PD-L1 [27]. Thus, we tested whether PD1 on the surface of engineered CDNVs could bind to cancer cell PD-L1 and block inhibitory signaling in NK cells. Control CDNVs or PD1+ CDNVs were incubated with HuCCT1 cells, then co-cultured with primary NK cells at 5:1 or 10:1 E:T ratios [28]. Pre-treatment with PD1+ CDNVs increased NK cell degranulation at an E:T ratio of 10:1, while enhanced cytotoxicity against cancer cells was observed at both the 5:1 and 10:1 ratios (Figure 10), similar to that observed with T cells. 

Collectively, these results indicate that CDNVs engineered to express PD1 could modulate biological interactions between PD1 and PD-L1, thereby enhancing anti-tumor T cell responses as well as stimulating innate anti-tumor immunity by improving the ability of NK cells to detect and destroy cancer cells. Further optimization of PD1+ CDNV formulations could help amplify their immunostimulatory properties.

## 4. Discussion

This study demonstrated the ability to engineer CDNVs to express desired surface proteins for targeted therapeutic effects. PD1-expressing CDNVs were generated from PD1-transfected A-MSCs, imparting selective PD1 surface expression on the membranes of the resulting nanovesicles. Compared to EVs derived directly from parental cells, these CDNVs offered major advantages in terms of yield, customizability, and cargo loading capacity. CDNVs can be generated from many different types of donor cells, including tumor cells, dendritic cells, and stem cells [6,29,30]. While conventional EV isolation methods typically yield low quantities, CDNVs generated from cells resulted in a higher yield of nanovesicles. This scalable approach also provides the ability to modulate the CDNV surface proteome through transfection of the source cells. Additionally, engineered CDNVs showed efficient loading capacity for therapeutic RNA and protein cargoes. Their ability to package and protect a range of nucleic acid and protein payloads supports their utility as delivery platforms. Together, these findings highlighted the major benefits of fabricating artificial vesicle mimetics compared to direct EV harvesting. This supports CDNVs as promising customizable nanocarriers engineered for targeted therapy and drug delivery applications where the use of conventional EVs may have limitations.

While CDNVs are promising alternatives to natural EVs for drug delivery, comprehensive toxicity assessments are imperative prior to their clinical translation. Safety assessments also allow for comparison and benchmarking with established standards for biologics and nanomaterials. Thus, safety assessments are essential to advance CDNVs toward clinical testing and evaluation of their potential as next-generation drug delivery vehicles. The studies performed herein using in vitro and in vivo models demonstrated an encouraging safety profile for CDNVs. No concerning adverse effects were elicited at efficient therapeutic doses. While long-term impacts remain to be evaluated, these findings provide evidence of the biocompatibility of CDNVs as cell-derived nanotherapeutics. Their lack of toxicity favors their advancement as promising drug delivery platforms. Moreover, the ease of rapid in vivo screening in zebrafish provides an efficient platform to evaluate various CDNV formulations and payloads to guide optimization and preclinical advancement, for example, with longer-term culture, repetitive dosing, and additional endpoints. 

PD1 display is an attractive engineering strategy for targeted nanotherapeutics. The PD1/PD-L1 immune checkpoint pathway is a ubiquitous therapeutic target across many different cancer types [31]. PD-L1 is overexpressed on many tumor cells, and binding to its receptor PD1 on immune cells leads to T-cell and NK cell inactivation leading to immune evasion [32]. Engineering nanovesicles to express PD1 on their surface enables specific targeting of tumor cells [26]. This allows for localized delivery of encapsulated drug payloads directly to the tumor microenvironment. PD1-engineered vesicles can also block PD1/PD-L1 interaction, reactivating the immune system’s anti-tumor functions. Thus, CDNV displaying PD1 has a multi-pronged immunomodulatory effect. Although EVs can be engineered to express PD1-targeting ligands or antibodies on their surface, their cumbersome isolation and limited yield have hindered their further development as therapeutics [31]. CDNVs generated by extrusion methods offer several advantages over naturally derived EVs that position them as promising therapeutic nanoplatforms. CDNVs can be produced in substantially higher yields and in more reproducible and scalable processes compared to EV isolation from MSCs or immune cells. Their lack of toxicity and customizable surface engineering enable tailored targeting and cargo loading that are not easily achievable with natural EVs. Specifically, this study found that CDNVs exhibited higher production yields, efficient incorporation of RNA/protein cargo, and no adverse effects in rigorous toxicity profiling. Surface engineering of CDNVs to display PD1 enabled specific targeting of PD-L1+ tumor cells and enhanced anti-tumor immune responses. Thus, CDNV has several advantages over EV in scalability, customizability, and cargo loading as promising PD1/PD-L1 targeted immunotherapies. Together, these findings showcase CDNVs as next-generation EV mimetics whose customization and scalability can overcome limitations in natural EV yield, reproducibility, and functionality for targeted therapeutic delivery.

## 5. Conclusions

In conclusion, this study demonstrates the potential of CDNVs as versatile nanoplatforms for targeted therapy and immunotherapy applications. While promising, further research is warranted to optimize large-scale production methods, evaluate long-term impacts, and refine techniques for controlled cargo release. Additional cell sources, surface proteins, and cargo can be explored to further enhance the efficacy of CDNVs for future therapeutic use across diverse applications, including cancer immunotherapy, regenerative medicine, and diverse organ-specific diseases. Enhancement and fine-tuning of RNA and protein therapeutic loading and release kinetics could maximize their potential for tailored delivery applications. Modifications of CDNVs with the incorporation of other cell-specific or other targeting ligands could expand their potential applications. Optimization of the protocol for scale-up and production within a GMP-compliant process that yields homogeneous populations of engineered CDNVs with relevant quality controls will be necessary. Overall, this study provides key proof-of-concept for the use of CDNVs as vectors for intracellular drug and gene delivery and a strong foundation to support continued investigation and optimization of CDNVs as next-generation therapeutic nanoplatforms.
